# The Pleiotropic Potential of BDNF beyond Neurons: Implication for a Healthy Mind in a Healthy Body

**DOI:** 10.3390/life11111256

**Published:** 2021-11-17

**Authors:** Maria Carmela Di Rosa, Stefania Zimbone, Miriam Wissam Saab, Marianna Flora Tomasello

**Affiliations:** 1 Department of Biomedical and Biotechnological Sciences, University of Catania, Via S. Sofia 64, 95123 Catania, Italy; mcdirosa@unict.it (M.C.D.R.); mirisaab@gmail.com (M.W.S.); 2 Institute of Crystallography, CNR, Via P. Gaifami 18, 95126 Catania, Italy; stefania_zimbone@libero.it

**Keywords:** BDNF, hypothalamus, mitochondria, exercise, metabolism, energy balance, pleiotropic, neurons

## Abstract

Brain-derived neurotrophic factor (BDNF) represents one of the most widely studied neurotrophins because of the many mechanisms in which it is involved. Among these, a growing body of evidence indicates BDNF as a pleiotropic signaling molecule and unveils non-negligible implications in the regulation of energy balance. BDNF and its receptor are extensively expressed in the hypothalamus, regions where peripheral signals, associated with feeding control and metabolism activation, and are integrated to elaborate anorexigenic and orexigenic effects. Thus, BDNF coordinates adaptive responses to fluctuations in energy intake and expenditure, connecting the central nervous system with peripheral tissues, including muscle, liver, and the adipose tissue in a complex operational network. This review discusses the latest literature dealing with the involvement of BDNF in the maintenance of energy balance. We have focused on the physiological and molecular mechanisms by which BDNF: (I) controls the mitochondrial function and dynamics; (II) influences thermogenesis and tissue differentiation; (III) mediates the effects of exercise on cognitive functions; and (IV) modulates insulin sensitivity and glucose transport at the cellular level. Deepening the understanding of the mechanisms exploited to maintain energy homeostasis will lay the groundwork for the development of novel therapeutical approaches to help people to maintain a healthy mind in a healthy body.

## 1. Introduction

Brain-derived neurotrophic factor (BDNF) is a member of the neurotrophin family along with the nerve growth factor (NGF), neurotrophin 3 (NT-3) and neurotrophin 4 (NT-4) [1]. BDNF is critically involved in neuronal development and synaptic plasticity; it promotes neuronal differentiation stimulating neurite outgrowth and synapses formation, and can prevent apoptosis [2].

For the crucial and physiological functions performed, the gene structure of BDNF is highly conserved throughout mammals and its transcription is tightly regulated and cell-type specific [1].

BDNF and its high-affinity receptor TrkB (tropomyosin-related kinase B) are widely expressed in the developing and mature central nervous system (CNS) and in many peripheral tissues, including muscle, liver and adipose tissue [3].

BDNF is synthesized as pro-BDNF and converted into mature BDNF (mBDNF) by furin and proconvertases within intracellular vesicles. Neurons are able to release either mBDNF or pro-BDNF in response to numerous stimuli. The ratio between pro-BDNF and mBDNF changes during distinct developmental stages and postnatal life [1,4,5,6]. When released, pro-BDNF is converted by the tissue plasminogen activator/plasmin system into mBDNF or may act as an independent ligand preferentially on the neurotrophin receptor p75 (p75^NTR^), a member of the tumor necrosis factor family of receptors. Although with a much lower affinity, mBDNF is also able to bind the p75^NTR^. Notably, TrkB and p75^NTR^ seem to mediate functionally antagonistic actions [1,2,7].

Once released, mBDNF forms stable homodimers and exerts its action locally, since the biochemical characteristics prevent the broad diffusion of the neurotrophin [1]. The binding of mBDNF to TrkB induces receptor dimerization and the autophosphorylation of tyrosine residues in the intracellular domain, which triggers a complex set of signaling cascades, specifically the tissue phospholipase C (PLC), phosphatidylinositol-3 kinase (PI3-K) and MAPK pathways. This results in the activation of specific transcriptional factors which in turn control the expression of proteins involved in plasticity, neuronal survival, cellular energy balance and mitochondrial biogenesis. BDNF also upregulates antioxidant proteins, modulates cytoskeletal dynamism and prevents apoptosis by promoting the expression of the anti-apoptotic Bcl-2 family members and inhibiting the pro-apoptotic ones [2,8].

This review deals with the intriguing topic of the BDNF involvement in energy homeostasis. In particular, we will discuss the most recent findings and theories hinting at a role for BDNF as a homeostatic factor regulating energy intake and expenditure in both the CNS and the peripheral metabolic organs.

## 2. The Role of BDNF in the Central Control of Energy Balance

Energy homeostasis is the result of a complex interplay between the brain and peripheral tissues. Neuronal circuitry in the hypothalamus and hindbrain receives and integrates peripheral signals related to hunger, satiety and energy storage in the body and elaborate specific responses regulating nutrient intake and energy expenditure. Therefore, to maintain energy balance, organisms have to evaluate changes in energy needs considering several factors such as physical activity and thermoregulation. Hormones such as insulin, leptin, PYY and ghrelin, allow this tightly regulated information exchange [9,10,11]. Alongside hormones, emerging players in the energy balance regulation are neurotrophins, most notably, BDNF and its receptor, both abundantly expressed in several regions of hypothalamus and hindbrain [3,12].

The involvement of BDNF in the central regulation of feeding was noticed earlier during investigations aiming at improving the knowledge about how BDNF regulates learning, memory, synaptic transmission and plasticity [11]. The intracerebroventricular (ICV) injection of BDNF in rats to evaluate the neurotrophic effect in the treatment of Alzheimer’s also induced appetite suppression and weight loss [13,14]. In mice models of diabetes and obesity, BDNF administration produced anorexigenic effects, reduced blood glucose and increased pancreatic insulin content [15,16]. These data were also confirmed in BDNF heterozygous mice in which about half of them exhibited a hyperphagic obesity; notably, chronic brain infusion of BDNF in obese BDNF-deficient mice was able to transiently reduce body weight [17]. BDNF involvement in the regulation of energy homeostasis was also reported in humans, in a young patient characterized by the early onset of obesity and hyperphagia along with developmental delays and other neurological defects. The young patient carried a heterozygous mutation in the NTRK2 gene, which encodes for the TrkB receptor. Namely, the mutation impinges on the activation loop of the TrkB catalytic domain (Y722C) leading to the loss of TrkB function [11,18]. Furthermore, mutations in the gene encoding BDNF are associated with eating disorders inducing obesity, hyperactivity as well as impaired cognitive functions. These data link BDNF to the regulation of energy homeostasis in humans [19,20,21] (Box 1 and Table 1).

Box 1 **Genetic studies in humans carrying BDNF mutations.**

Genetic disorders causing BDNF haploinsufficiency or TrkB inactivation provide direct evidence for the understanding of the role of BDNF in the regulation of energy balance in humans. A deficit in BDNF expression or in its signalling may contribute to weight gain and cognitive impairment which vary in phenotypic severity [22,23].

Gray et al. identified a chromosomal inversion encompassing BDNF gene in an 8-year-old girl with hyperphagia, severe obesity, hyperactivity and cognitive impairment. The chromosomal inversion affected BDNF expression with a loss of function without disrupting the sequence of the gene itself [19]. An additional study reported a BDNF haploinsufficiency in a cohort of patients with WAGR syndrome, a rare genetic disorder caused by contiguous gene deletions on chromosome 11p13 region of varying size. Since the BDNF gene is located in the chromosomal locus 11p14.1, approximately half of the patients with WAGR syndrome had a heterozygous BDNF deletion. In these subjects, BDNF haploinsufficiency was significantly associated with a rise in body mass index, hyperphagia and reduced BDNF levels in serum along with neurocognitive impairments [20]. Similarly, low levels of serum BDNF were reported in patients with metabolic and eating disorders [24,25].

Several genome-wide association studies have strengthened these findings identifying BDNF single nucleotide polymorphisms (SNPs) linked to an increased risk of developing obesity in humans. The most commonly studied BDNF SNP is the G196A variant which causes the substitution of valine with methionine in the pro-BDNF position 66 (Val66Met). The amino acid change affects the intracellular trafficking and packaging of pro-BDNF impinging the activity-dependent secretion of mBDNF [22,26,27,28]. A rare de novo missense variant in BDNF and seven NTRK2 mutations along with three variants previously reported [23] have also been functionally characterized by Sonoyama et al. Clinical data collected from carriers of BDNF/TrkB variants showed in addition to severe obesity, a spectrum of neurobehavioral disorders such as hyperactivity, learning deficit and shot-term memory impairment [29]. Interestingly, a latest study reports an atypical Charcot–Marie–Tooth disease type 2Q phenotype with obesity likely related to the mutation identified in the coding region of the NTRK2 gene [30].

All things considered, there is clear evidence of the crucial role of BDNF in modulating body weight and energy homeostasis, although further studies are needed to better elucidate the BDNF involvement in these syndromes (Table 1).

**Table 1:** Clinical studies on common and rare variants in BDNF and NTRK2.

Gene	Mutations	Phenotypic Features	Reference
BDNF	11p inversion; haploinsufficiency	Severe obesity, hyperphagia, impaired cognitive function, hyperactivity	[19] Gray, J. et al., 2006 [22] Han, J.C., 2016
	Deletions including the 11p14 BDNF locus among patients with WAGR syndrome; haploinsufficiency	Obesity, hyperphagia, lower levels of serum BDNF	[20] Han, J.C. et al., 2008 [22] Han, J.C., 2016
	Intronic SNP: rs12291063 CC genotype	Obesity	[21] Mou, Z. et al., 2015
	SNP rs6265 commonly known as G196A => Val66Met	Susceptibility to obesity, several psychiatric conditions including eating disorders	[25] Rosas-Vargas, H. et al., 2011 [26] Vidović, V. et al., 2020 [27] Ieraci, A. et al., 2020 [22] Han, J.C., 2016
	Missense mutation E183K	Severe obesity and moderately learning difficulties	[29] Sonoyama, T. et al., 2020
NTRK2	Missense mutation Y722C	Severe early-onset obesity, hyperphagia, developmental delay	[18] Yeo, G.S.H. et al., 2004
	Missense mutations: I98V, P660L, T821A	Severe obesity, developmental delay	[23] Gray, J. et al., 2007
	Missense mutations: P204H, R691H, R696K, S714F, R715Q, R715W, P831L	Severe obesity, hyperactivity, maladaptive behaviours and impaired short-term memory	[29] Sonoyama, T. et al., 2020

In the hypothalamus, BDNF is synthesized in several regions participating in metabolic homeostasis, including the ventromedial hypothalamic nucleus (VMH), the dorsomedial hypothalamic nucleus (DMH), the paraventricular nucleus (PVH) and the lateral hypothalamic area (LH). The arcuate nucleus (ARC), a crucial hypothalamic center controlling energy balance, does not seem to be involved in BDNF synthesis but in its function [3,11]. In fact, TrkB is expressed in the ARC, which in turn, displays two functionally different populations of neurons: (i) cells producing the anorexigenic polypeptides cocaine- and amphetamine-regulated transcript (CART) and proopiomelanocortin (POMC), a precursor of α-melanocyte stimulating hormone (α-MSH), the ligand of melanocortin receptor 4 (MC4R), and (ii) cells producing the orexigenic neuropeptide Y (NPY) and the agouti-related protein (AgRP), an antagonist of MC4R [10,12]. Metabolic and nutritional signals mediated by peripheral factors are integrated in this region which is connected to other hypothalamic nuclei crucial in the control of feeding. Notably, BDNF seems to be essential in promoting axonal projections of TrkB expressing neurons from ARC to the PVH and DMH [31]. An earlier study also showed a link between the local translation of BDNF in hypothalamic neuronal dendrites and the activity of leptin and anorexigenic hormone released by adipose tissue [32,33].

The VMH represents the principal region where BDNF is produced in response to finely tuned stimuli integrated from nutritional cues such as glucose, leptin or fasting. Unger and collaborators demonstrated that glucose administration in adult mice induced an increase in both BDNF and TrkB levels in VMH and that the deletion of the BDNF gene in the VMH and DMH produced hyperphagic obesity without changing energy output and locomotion. Thus, the VMH and DMH have been pinpointed as important sources of BDNF for the regulation of satiety and appetite suppression [34]. It was also shown that BDNF expression in VMH is upregulated in response to leptin [35] and modulated by MC4R signaling [36]. In particular, the evidence that: (i) a MC4R agonist largely raised BDNF mRNA levels in the VMH following a period of food deprivation and (ii) the brain infusion of BDNF restored a normal feeding behavior in mice deficient of MC4R signaling and fed with a high-fat diet (HFD) suggested the presence of circuitry in which BDNF represents a downstream effector of MC4R signaling [36,37]. To date, the mechanism regulating the synthesis of BDNF is still unclear. BDNF expression could be directly mediated by specific cues, as described above, and/or indirectly triggered via the action of relevant stimuli acting on circuits connecting hypothalamic nuclei each other [11,35].

Similar results were achieved in the dorsal vagal complex (DVC) of the hindbrain. Studies in adult rats provide evidence that BDNF delivery in the DVC negatively regulates food intake and that BDNF/TrkB signaling could mediate the action of melanocortins and could contribute to the leptin anorexic effect [38,39,40].

The key role of BDNF in regulating energy balance was also investigated in the PVH. In particular, the injection of BDNF in this region caused a loss of body weight in rats by reducing food intake and promoting energy expenditure as a consequence of an increased resting metabolite rate, likely associated with the thermogenic effect of the uncoupling protein 1 (UCP1) in the brown adipose tissue (BAT) [41,42]. Alongside the regulation of food intake, the involvement of BDNF in energy expenditure may also be mediated through the control of BAT thermogenesis and locomotor activity. In particular, by assessing the effect of the BDNF ablation in the PVH, An et al. revealed the presence of discrete neuronal populations associated with different functions, for example, BDNF neurons in the anterior PVH related to hyperphagia and reduced locomotor activity and BDNF neurons in medial and posterior PVH promoting thermogenesis through polysynaptic connections with the BAT [43]. In a follow-up study, the same authors, using a projection-specific gene deletion approach, accurately investigated the action site of PVH neurons expressing TrkB and identified neuronal networks with the VMH and lateral parabrachial nucleus (LPBN) involved in appetite suppression [44].Energy consumption mediated by thermogenesis and physical activity was furthermore induced by the activation of neurons that express TrkB in the DMH. This recent study revealed distinct neuronal populations expressing TrkB which create neurocircuitry by projections to several brain regions (raphe pallidus, PVH and preoptic area) to accurately manage energy expenditure and food intake [45]. The more significant studies investigating the role and the effects of BDNF in the central control of energy balance are summarized below (Table 2).

## 3. BDNF in Energy Expenditure

As introduced in the previous paragraph, BDNF plays a key role in the management of energy balance, a tightly regulated equilibrium between energy intake and expenditure. In an organism, the total energy expenditure (TEE) has as a major component in the basal metabolic rate (70%), which is the energy employed for the physiological operation of cells and organs under resting conditions. The energy expended in physical activity represents 20% of TEE and includes both volitional activity (such as exercise) and non-volitional activity such as thermoregulation, spontaneous muscle contractions and posture maintenance. The remaining part of TEE (10%) is spent in digestion, absorption, and sympathetic nervous system activation after the ingestion of a meal, in other words, it is the energy required to access the energy of the nutrients [46]. The peripheral tissues and organs, including the liver, the pancreas, the adipose tissues and the gastrointestinal tract, all build a highly complex system that contributes to the control of the energy balance in response to changes in the feeding state and the size of energy storage. The signals are integrated by neurons in the hypothalamus, resulting in an anorexigenic or orexigenic effect.

In the following paragraphs, we discuss the function of BDNF in both volitional and non-volitional physical activities.

### 3.1. BDNF in Thermoregulation

Thermogenesis is considered a non-volitional physical activity relevant to keeping body temperature within certain boundaries, even when the surrounding temperature is very different. Defined as the dissipation of energy to produce heat, thermogenesis occurs in BAT and skeletal muscle. BAT thermogenesis is controlled by the sympathetic drive, which is connected polysynaptically with neurons in the hypothalamus.

Several studies have reported the thermogenic effects of BDNF that can act both on hypothalamic neurons or on BAT. In mice, the ablation of BDNF results in deficits of BAT-mediated thermogenesis, impairments of the body temperature in response to cold, and finally, in obesity. At the molecular level, this is associated with the reduced expression of UCP1 and of the peroxisome proliferator-activated receptor-gamma coactivator (PGC1α), both involved in thermogenesis. UCP1 is a mitochondrial proton carrier that uncouples the respiratory chain from adenosine triphosphate (ATP) synthesis, allowing heat generation. PGC1α is a transcription factor involved in energy metabolism (both carbohydrate and lipid metabolism) by regulating mitochondrial biogenesis [47].

Long-term peripherally administered BDNF increased body temperature and oxygen consumption [16] while hypothalamic injection of BDNF increased UCP1 mRNA and protein in BAT [48]. Furthermore, BDNF overexpression is suggested to induce the switching of white adipose tissue (WAT) to the BAT phenotype through sympathetic neuron activation in response to environmental stimuli [49]. The exogenous administration of BDNF regulates BAT thermogenesis through the brain control of sympathetic neurons. While BDNF infusion into the PVH increases energy expenditure coupled with an elevation of UCP1-mediated thermogenesis [41], energy expenditure only, and not UCP1 levels, are increased when BDNF is injected into the VMH [50]. In the murine DMH, neurons expressing BDNF are quickly activated by cold temperatures, and their activation is sufficient to increase body temperature, energy expenditure and physical activity [51]. Considering that BAT thermogenesis is the main component of energy expenditure in these animals, rodents use β-oxidation of fatty acids in BAT to maintain body temperature. Interestingly, although BAT was believed to be absent in adult humans, recent research reported a significant amount of metabolically active BAT involved in the regulation of thermogenesis in humans [52].

Overall, the findings discussed above provide evidence that BDNF has a role in the regulation of thermogenesis, and that this function is evolutionarily conserved.

### 3.2. BDNF in Volitional Physical Activity

Volitional physical activity consists of bodily movement produced by skeletal muscles both in daily life and in planned activities such as exercise or training. Both require energy expenditure and induce expression of BDNF in the skeletal muscle and in the brain, which performs its function in local and peripheral tissues. Many years of research have demonstrated the benefits of exercise for the brain. These benefits depend on BDNF release and result in the improvement of cognitive function in both animals and humans [53,54,55].

**Figure 1:**
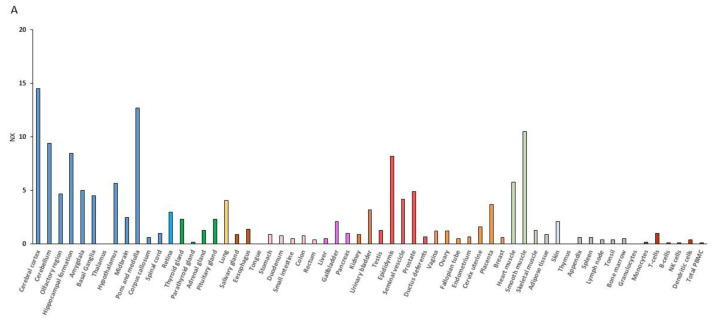

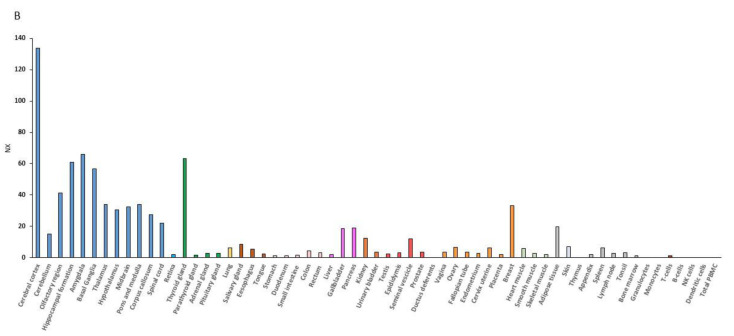
BDNF (**A**) and NTRK2 (**B**) RNA expression overview. NX indicates consensus normalized expression levels for 55 tissue types and 6 blood cell types, obtained by combining the RNA-data from three transcriptomics datasets (HPA, GTEx and FANTOM5) using the internal normalization pipeline. Each color indicates different groups consisting of tissues with common functional characteristics (source: *Tissue expression of BDNF—Summary—The Human Protein Atlas; Tissue expression of NTRK2—Summary—The Human Protein Atlas*).

RNA expression data have demonstrated that the muscle is second to the brain for the BDNF expression level (Figure 1). BDNF mRNA expression is indeed increased upon contraction of muscular fibers, and the muscle can secrete BDNF into circulation [56,57]. Additionally, the BDNF receptor, TrkB, is upregulated in animals put under rigorous physical training [58,59] or after the BDNF overexpression [60].

Accordingly, in mice, Johnson et al. found that physical training leads to an increase in specific hippocampal BDNF expression, [61], resulting in stimulation of cognition and memory improvement [62].

However, evidence from human studies is much less direct. Several data indicate that physical activity stimulates the brain to produce and release more BDNF in order to improve muscle repair [63]. For instance, Rasmussen et al. by measuring the plasma BDNF levels during physical activity in humans, observed that BDNF increases two-to-three-fold during the exercise phase and begins to lower at rest 1 h later when the exercise has stopped. They calculated that the brain contributed to 70–80% of the circulating BDNF, either at rest or during the physical exercise, which suggest that through BDNF the brain participates to the tissue regeneration after muscle injury [63].

In a similar manner, Erickson and collaborators show that higher serum BDNF levels due to physical activity associates with increased hippocampal volume in the elderly. Based on a randomized controlled trial of 120 older adults, they found that aerobic exercise training increased hippocampal volume by 2%, reversing age-related volume shrinkage. This correlates with higher serum levels of BDNF, suggesting again that physical exercise, through BDNF, stimulates cell repair and regeneration in several tissues [64].

Interestingly, another study highlighted the finely tuned regulation of BDNF release. It was reported that during physical training the amount of serum BDNF plateaued at longer running distances [65] revealing a physiological saturation mechanism that protects against potential excitotoxicity due to an excess of BDNF [66].

In the skeletal muscle, BDNF induced by exercise affects the physiology and morphology of fibers and is involved in the recovery of damaged muscles. These properties lead to consider BDNF as a myokine, although its role in neuromuscular physiology regulation in vivo remains unclear [67,68].

In adult skeletal muscle, BDNF expression is found in myogenic progenitors known as satellite cells [69]; in healthy muscle, satellite cells are mitotically quiescent while they become activated in response to injury, triggering proliferation and differentiation to repair damaged fibers. In order to study the involvement of BDNF in muscle damage repair, Clow and Jasmin generated a mouse model in which BDNF is specifically depleted from skeletal muscle cells [70]. Moreover, after muscle injury BDNF expression is up-regulated when activation and proliferation of satellite cells occur [71]. Another study reports the up-regulation of BDNF in the extensor digitorum longus and tibialis anterior muscles of mice during the development period [72]. In this work, the authors demonstrated that the administration of BDNF prevented the loss of motor units (or the disappearance of muscle fibers) after neonatal nerve injury and contributed to the maintenance of muscle mass [72]. The differential expression of BDNF in skeletal muscles, according to diverse physiological or pathological conditions, strengthens the notion that BDNF actively contribute to tissue recovery [73].

The influence of BDNF on skeletal muscle goes beyond regeneration and differentiation. Some evidence has demonstrated a role for BDNF in the energy metabolism and specification of skeletal muscular fibers. Delezie et al. have observed that muscles depleted of BDNF show higher succinate dehydrogenase activity, a mitochondrial enzyme involved in oxidative phosphorylation. Moreover, they demonstrated that the lack of BDNF contributes to the transformation from type IIB fibers, which have a low oxidative metabolism and are less efficient to fatigue, to type IIX fibers with a high oxidative capacity and are relatively efficient to fatigue. Conversely, BDNF overexpression increases the fast-type muscle gene expression, typical of type IIB fibers, such as Baf60c and Tbx15, and glycolytic markers, such as Serca1 and its coregulator Mlrn [74]. The involvement of BDNF in the glycolytic fiber-type specification was confirmed by Yamanaka et al. [75]. In this way, the BDNF-induced glucose uptake contributes to whole-body metabolism linked to the glycemic control since skeletal muscle is considered the most important storage of glucose upon insulin stimulation. The involvement of BDNF in thermoregulation and volitional physical activity is summarized in Table 3.

### 3.3. BDNF, Mitochondria and the Regulation of Cellular Bioenergetics

While it is well recognized that the exercise-induced BDNF has implications on neuronal metabolism, [76] how BDNF modulation during exercise can influence specific aspects of cognitive function is poorly understood.

Research on the topic pointed out that, when upregulated due to physical exercise, BDNF can affect mitochondrial metabolism in the CNS [2]. In the hippocampus, in the cortex and in the cerebellum, the upregulation of BDNF stimulates PGC-1α expression which is involved in mitochondrial biogenesis and modulates autophagy with many beneficial effects [77,78,79,80]. Similar to many other genes involved in vital processes for the cell and the organism, PGC1α expression is controlled by the transcription factor CREB (cyclic AMP response element-binding protein) via phosphorylation. In particular, BDNF induces Ca^2+^ influx through the TRPC (transient receptor potential C) channels. The consequent intracellular Ca^2+^ rise, activates the Ca^2+^/calmodulin-dependent protein kinase (CaMK), resulting in CREB phosphorylation/activation. Phospho-CREB, in turn, stimulates PGC-1α expression, which increases the mitochondria content. Thus, BDNF indirectly ensures more energy substrates (ATP and NAD+) to support the adaptive plasticity and the formation of synapses [81]. Interestingly, NAD+ induces sirtuin-1 (Sirt1) expression, a deacetylase that can activate the transcription factor FOXO3a, resulting in the production of the mitochondrial antioxidant enzyme MnSOD (manganese superoxide dismutase) [80]. This evidence suggests that BDNF is also involved in antioxidant defense. Moreover, the exercise-dependent PGC-1α-activation in the brain stimulates the expression of FNDC5 [82]. FNDC5 is a myokine, cleaved and secreted as irisin during exercise, which mediates the metabolic benefits of exercise [83]. Notably, FNDC5 was shown to regulate BDNF release in the hippocampus and secreted irisin induces the “browning” of adipose tissues. This reinforces the idea of the wide beneficial effects of exercise on the entire organism.

Exercise can differently influence young and old organisms. Gusdon et al. analyzed the mitochondrial functionality and the pathways regulating mitochondrial biogenesis both in young and old mice. After three weeks of physical training, the functionality of the mitochondrial electron transport chain complex I, but not complex II, is improved in old mice. The improvement in mitochondrial functionality is limited to old mice since young mice did not show differences following the training. This was associated with increased BDNF levels. Furthermore, in old mice, cortical neurons do not exhibit increased brain mitochondrial protein expression or mitochondrial biogenesis following exercise, but rather elevated DRP1 levels (dynamin related protein-1), suggesting an enhanced mitochondrial transport or turnover by fission. Mitochondrial fission is indeed needed for the effective autophagic clearance of damaged mitochondria [84]. The direct impact of BDNF on complex I functionality is described by Markham et al. They found BDNF action to be specific for brain mitochondria, with no effect in liver mitochondrial preparations [85]. Moreover, the authors confirmed that the improvement of mitochondrial respiration is reached through the MEK-MAPK pathway [86]. Thus, the specific effect of BDNF on complex I activity, may have marked implications for neurodegenerative and psychiatric diseases associated with impaired mitochondrial metabolism.

In the hippocampus, the inhibition of BDNF activity during exercise training decreases the mRNA levels for AMPK (AMP-activated protein kinase), uMtCK (ubiquitous mitochondrial creatine kinase), UCP2 (uncoupling protein 2), ghrelin, and IGF-I (insulin-like growth factor-I) [87]. These proteins normally increase after exercise and are relevant in the regulation of bioenergetics. AMPK increases the cellular energy supply by switching on catabolic pathways to generate ATP and shutting off processes that dissipate it, through the phosphorylation of metabolic enzymes. The mitochondrial phosphocreatine uMtCK, can buffer the elevated ATP consumption by donating its phosphate group to ADP, providing a mechanism to protect neurons during periods of increased energy demand. UCP2 is an isoform of the mitochondrial uncoupling protein, expressed in many different tissues, including the kidney, liver, gastrointestinal tract, brain, and skeletal muscle. UCP2 enhances energy expenditure and protects neonatal neurons from excitotoxic cell death, inhibiting reactive oxygen species production and preventing mitochondrial dysfunction. IGF1 is shown to protect cultured hippocampal neurons from serum deprivation-induced cell death using similar downstream pathways [76,87]. Although the IGF-I receptor is abundantly expressed in the hippocampus, IGF-I can also be produced in peripheral tissue, such as in skeletal muscle and liver in response to exercise. Finally, ghrelin is generally secreted from the oxyntic glands of the stomach [88] in response to energy restriction and/or depletion.

## 4. The Role of BDNF in the Peripheral Control of Energy Balance

In the previous paragraphs, we have discussed the BDNF involvement in the regulation of energetic metabolism and the way it is achieved through the modulation of the mitochondrial function, related to physical exercise. Alongside these concepts, BDNF seems to actively participate in nutrient uptake control in different types of cells, thus defining the differentiation of the given cell type toward a distinct physiological function.

Energy requests, both in the basal state and during exercise, are covered by two major substrates: glucose and free fatty acids (FFA). Conversely, protein oxidation occurs for the protein taken with the diet and contributes 10% to 15% of TEE. Glucose and FFA can be considered interchangeable as intermediate metabolites in most tissues, but the brain relies almost exclusively on glucose metabolism [46].

During postnatal brain development, BDNF signaling increases glucose and amino acid uptake, playing a determinant role in the response to the increased energy demand and protein synthesis associated with neuronal differentiation. In fact, in the brain, 50% of the total energy consumption is used to restore ion gradients and resting membrane potentials by Na+/K+-ATPase [89] useful to synaptic plasticity and electrical stimulation. Notably, in cortical neurons, 20 min BDNF exposure increases GLUT3 (neuronal glucose transporter) mRNA and protein levels, thus enhancing glucose utilization by increasing its uptake. This effect requires TrkB receptor activation and the PLC signaling pathway and is specific for cortical neurons since it is not described in cortical astrocytes [90].

BDNF participates in the regulation of peripheral energy metabolism by directly acting on those cells which are strictly involved in the glucose homeostasis such as pancreatic β cells and hepatocytes [91].

In peripheral tissues, glucose homeostasis is under the regulation of insulin signaling. In fact, in the liver of diabetic mice, BDNF treatment enhances the tyrosine phosphorylation of the insulin receptor which in turn trigger PI3-K signaling [92,93]. The reduction in blood glucose levels is associated with an increase in the number and total area of pancreatic islets and with an increase in secretory granules in β-cells [94]. It has been demonstrated that the synergistic effect of BDNF and glucose levels induces insulin release from pancreatic β-cells. In mice, the synergistic effect happens when blood glucose levels are high; in humans, insulin is released from islets, even in the presence of lower blood glucose levels. This is likely due to the intrinsic differences between mouse and human β-cells. Notably, their sensitivity to glucose as well as the expression of different ion channels, might influence β-cell sensitivity to intracellular Ca^2+^ levels induced by BDNF/TrkB signaling [57].

Furthermore, glucagon levels seem to be influenced by BDNF. Infusion of BDNF into the rat brains results in decreased glucagon levels in the portal vein. This effect is abrogated by the denervation of pancreatic efferent nerves [95]. Moreover, it is observed that the intraportal administration of GLP-1 (glucagon-like peptide 1) increases BDNF levels in the pancreas and reduces glucagon secretion. GLP-1 receptors are expressed in the pancreas, muscle, liver and also in neurons throughout the brain. The activation of GLP-1 receptors results in cyclic AMP production and in the activation of CREB which is known to induce BDNF expression. Similar to GLP-1, BDNF signaling increases glucose uptake by liver, skeletal and cardiac muscle cells [75].

BDNF might also be considered as anadipokine since it is expressed in both BAT and WAT [96,97]. In particular, significant changes in BDNF and NTRK2 expression are observed in the adipose tissue of obese mice (NTRK2 is downregulated by adipocytes, in contrast, BDNF is upregulated by other cells) implying that BDNF may have a role in the regulation of systemic metabolism [98] Other studies, performed in rats, demonstrated the activation of BDNF and TrkB-expressing neurons located in sympathetic outflow circuitry that ultimately innervate WAT, the tissue where lipolysis is stimulated. This sympathetic activation is responsible for the increase in circulating FFA and glycerol concentrations and for the decrease in body fat mass [99].

All these literature data allow the consideration of BDNF as a metabolic modulator that coordinates the adaptive response of the brain and the body to fluctuations in energy intake and expenditure; for this reason, it was defined as “metabokine”, meaning a pleiotropic signaling molecule [100].

In hypothalamic neurons, BDNF influences food intake through the activation of mTORC1 (mTOR complex 1). Similar to insulin, BDNF binds tyrosine kinase receptor, thus activating the PI3-K/Akt pathway [28,101,102]. The activation of mTORC1 stimulates protein synthesis and lipid biosynthesis resulting in cellular mass gain and reduced food intake needs. Notably, it is demonstrated that BDNF can act through different signal transduction pathways, mTOR and AMPK, although they work oppositely (i.e., when intracellular energy is abundant, mTOR activity is increased and AMPK activity is decreased, and vice versa) [103,104]. The types of signaling pathway activated depend on the tissue where BDNF acts. In myotubes and in contracting muscle BDNF promotes catabolic pathways, increasing β-oxidation through AMPK signaling that activates acetyl coenzyme A carboxylase (ACCβ) and consequently enhances fat oxidation and inhibits fat synthesis [56,67,105]. Similarly, in hepatocytes, BDNF activates the same molecular mechanism but this results in increased fatty acid oxidation, glycogen storage and inhibition of gluconeogenesis [106].

## 5. BNDF Involvement in Neurodegenerative Disorders

It is known that the pathos-physiological modifications associated with several neurodegenerative diseases begins decades before the emergence of clinical symptoms. One of these changes is the impairment in the brain energy metabolism. The brain, indeed, has high energy requirements, as it employs most of the glucose for the maintenance of synaptic functions and of neuronal resting potentials. As broadly discussed throughout this manuscript, among other functions, BDNF is closely involved in the regulation of energy balance, and BDNF levels are influenced by physical activities. Alongside being essential for the survival and phenotypic maintenance of mature, fully developed neurons, BDNF is recognized to modulate several neuronal functions, such as axonal growth, long-term potentiation, which is pivotal for the development of learning and memory. Therefore, it is not surprising that BDNF is implicated in several neurodegenerative diseases, including Alzheimer’s (AD), Parkinson’s (PD), Huntington’s (HD) diseases and other neuropsychiatric disorders [107,108].

The first evidence implicating BDNF in AD and PD dates back to the 1990’s, and for both pathologies, reduced levels of mRNA or proteins were described either in postmortem brains of humans or mice models [109,110,111,112]. Lower BDNF mRNA expression was reported in the hippocampus, neocortex, in the Meynert nucleus basalis, all of which are regions selectively vulnerable to the degeneration in AD [110]. Reduced BDNF levels were also associated to the presence of neurofibrillary tangles, a hallmark of AD [113]. Accordingly, the withdrawal of BDNF in cultured hippocampal neurons or BDNF depletion in mice also resulted in differential expression of genes implicated in AD. More importantly, genetic delivery of BDNF in primate and rodent models of ADreduce synaptic loss and improve learning and memory formation [114].

As in AD, reduced expression of BDNF mRNA and protein are found in dopaminergic neurons of the substantia nigra, a region of the brain where PD-affected neurons are localized [110,115,116]. This notion was confirmed when many classical features of animal PD models were reproduced by blocking the BDNF expression in the substantia nigra of rats [117]. Accordingly, Wnt-BDNFKO mice, completely lacking BDNF in the midbrain and hindbrain, show a persistent reduction of dopaminergic neurons in the substantia nigra [118]. In addition, reduced BDNF production is closely associated with pathogenic mutations in α-synuclein in familial PD [119,120].

The assessment of BDNF levels in human postmortem cerebral cortex samples indicated that BDNF production was also impaired in the brains of HD patients [121,122]. Several works confirmed the reduction of BDNF levels in a large panel of huntingtin (Htt) knock-out mice, indicating that a decrease in cortical BDNF levels occur early in the disease and that it is partly due to the lower stimulatory activity of wild type Htt. Indeed, the increase or decrease in wild-type Htt levels in mouse models augments or reduces, respectively, the transcription from the BDNF promoter [123,124]. Accordingly, Htt is thought to play a role in the transport and activity-dependent release of BDNF [123,125]. Thus, mutations in the Htt protein result in “loss of function”, greatly affecting BDNF levels in striatal neurons.

BDNF is reported to slow the progression of motor neuron atrophy in an animal model of amyotrophic lateral sclerosis (ALS) [126], and the TrkB agonist 7,8-dihydroxyflavone (7,8-DHF) improved motor neuron deficits in the superoxide dismutase 1 (SOD1G93A) ALS mouse models [127].

Recent clinical studies have also demonstrated an association between low levels of BDNF and depressive disorders. Accordingly, BDNF infusion produces anti-depressive-like effects in the mouse midbrain of depression mice models [128]. Finally, in the hippocampus, prefrontal cortex, anterior cingulate cortex and superior temporal gyrus of schizophrenia patients, BDNF mRNA and protein levels have been found to be lower than in the controls [129,130,131].

The above-mentioned findings, which demonstrated that levels of neurotrophins influence the progression of neurodegenerative disorders, are the rationale for developing therapeutic approaches based on the modulation of BDNF levels.

The first clinical trial considering the application of BDNF infusions in neurodegenerative diseases was performed in ALS patients but failed to demonstrate a statistically significant effect of BDNF on patients survival [132,133]. It is possible that the poor pharmacokinetics associated with the intact protein, the BDNF short in vivo half-life, the limited diffusion and low penetrability of the blood-brain barrier has hindered progress towards a therapeutic strategy. Consequently, since pharmacological treatments are not available, other remedies are found to delay the course of the disease: physical exercise, enriched environment, hormonal balance (i.e., steroid hormones such a cortisol and testosterone) and nutritional intervention (i.e., fasting, low-calorie intake, low-carb diet, selective nutrient intakes). Among them, epidemiological studies have found that physical activity reduces the risk of AD and dementia by 45% and 28%, respectively [134] and are capable to rescue BDNF levels [135]. In agreement with these results, preliminary findings indicate that in healthy individuals the deposition of plaque/tangle in the brain inversely correlate with an healthy life style (normal body weight, regular physical activity, and healthy diet) [136].

The research on neurodegenerative diseases discussed above, strengthens the concept that BDNF levels are influenced by physical activities. However, the underlying molecular mechanisms explaining these findings are unknown. Based on findings from animal models, Mattson proposed that physical activity and intermittent energy restriction can together hinder neurodegenerative processes and improve brain function supporting the neuronal adaptive stress response including DNA repair, neurotrophic signaling, mitochondrial biogenesis [137]. Physical exercise downregulates Bax and neuro-inflammatory cytokines in the hippocampus [138,139], reduces chronic oxidative stress and promotes mitochondrial biogenesis. Porrit and collaborators [117] reported that BDNF overexpression decreases the expression of PINK1, an integral protein which governs mitochondrial quality control [140], and restores the activity of key enzymes (complex I, complex II+III) in the mitochondrial respiratory chain. Others have shown that the pathways by which exercise-induced neuronal BDNF might involve the co-activation of PGC-1α, leading to the activation of fibronectin type III domain-containing protein 5 (Fndc5) gene expression [82]. Accordingly, exercise is known to enhance the activities of BDNF regulating transcription factors CREB and NF-κB [141] which can act in cooperation with FNDC5. On the other hand, several reports indicate that factors enhancing glucose uptake and glycolytic flux (e.g., Wnt3a) or regulating mitochondrial functions could be beneficial in AD, PD, ALS and in other neuropathological conditions [142].

From a unitary perspective, brain metabolism is intended to equate oxygen consumption to glucose utilization. However, the concept of increased non-oxidative glucose consumption during physiologic neural activity has recently gained a lot of consideration. Enhanced aerobic glycolysis and increased lactate production are recognized as common properties of invasive cancers and its up regulation in cancer results in the suppression of apoptosis. This phenomenon, termed the Warburg effect, is progressively being recognized as well in the CNS as an adaptive response which would provide selective advantages for neuronal survival. Our group previously reported on a neuro-protective effect of monomeric Aβ in vitro which is directly connected to the regulation of glucose utilization in neurons [143]. This effect mediated by the IGF-IR and the ensuing PI3K pathway, results from the stimulation of the CREB target genes including BDNF [144]. Most recently, we have demonstrated that in response to the inhibition of oxidative phosphorylation, cultured cortical neurons increased aerobic glycolysis. This increase depends on the stimulation of the PI3K pathway and involves the activation of AKT, a master regulator of survival/apoptosis which targets various proteins including hexokinase (HK). HK is mainly associated with the outer mitochondrial membrane [145]. Mitochondrial-bound HKI supports neurons and the HK-released from mitochondria decreases in enzyme activity and triggers apoptosis in cells.

On these premises, we speculate that, by stimulating specific pathways, BDNF prompts neurons to exploit either oxidative phosphorylation or aerobic glycolysis in order to quickly fuel neurons with the necessary energy to properly absolve their functions. This would represent a physiological homeostatic mechanism to ensure synaptic plasticity. Consequently, a reduction in BDNF and other neurotrophins, as occurring in aging and in diverse neuropathological conditions, might impair the neuronal ability to cope with transient needs in energy provision.

## 6. Conclusions

The incidence of obesity and metabolic diseases among children, adolescents and adults is progressively increasing across the globe. Derived from an imbalance between food intake and energy expenditure, metabolic disorders augment the risk of cardiovascular and neurodegenerative diseases, negatively affecting people’s lifestyles.

**Figure 2:**
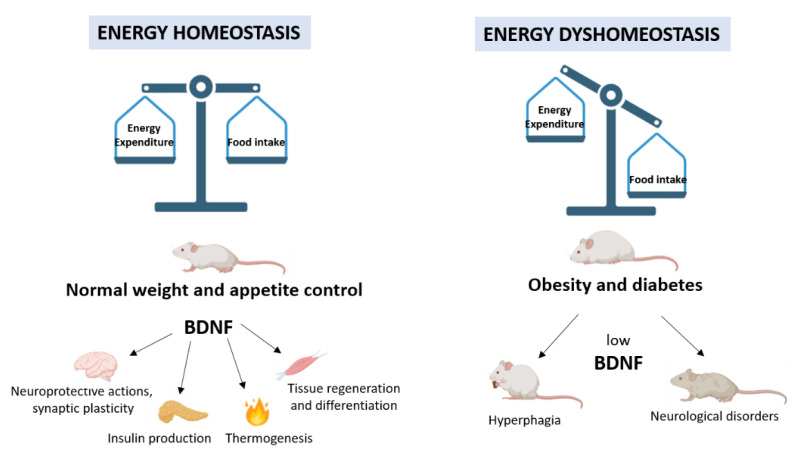
Schematic drawing of BDNF involvement in energy homeostasis. BDNF modulates numerous pathways related to food intake and weight control not only via the brain but also via peripheral neurons and tissues involved in preserving energy balance. When food intake exceeds energy expenditure, pathological conditions (obesity and diabetes) may occur.

Strong evidence from genetic and pharmacological studies highlights the crucial role of BDNF in controlling energy homeostasis. As a metabolic modulator, BDNF coordinates the adaptive response to fluctuations in energy intake and expenditure mediating signal transfer through a complex network between the CNS and peripheral tissues (Figure 2).

The energy balance can be maintained through a healthy lifestyle including diet and volitional physical activity. Interestingly, a large amount of data supports the involvement of BDNF in the realization of beneficial effects induced by physical exercise. The identification of circuitry which integrates signals from food intake and energy expenditure and the elucidation of the mechanisms by which BDNF acts on them will lay the groundwork in building a healthy and balanced life and deepening the comprehension of human metabolic diseases. The resulting knowledge could have important implications for human health, leading people to maintain healthy bodies and minds, and encouraging the identification of novel strategies for treating metabolic disorders.

## Figures and Tables

**Table 2:** The more significant studies investigating the role and the effects of BDNF in the central control of energy balance in rodents’ models.

Animal Model	Intervention/Stimuli	Effects	Reference
Wistar rats	Chronic intraventricular administration of BDNF and NGF	Reduction in weight gain	[13] Lapchak, P.A.; Hefti, F., 1992
Long-Evans rats	ICV BDNF infusion (lateral ventricle)	Appetite suppression and weight loss	[14] Pelleymounter, M.A et al., 1995
C57BL/KsJ-db/db mice (obese diabetic mice)	BDNF central (ICV) administration	Reduction in blood glucose and increase in pancreatic insulin	[15] Nonomura, T. et al., 2001
C57BL/KsJ-db/db mice (obese diabetic mice); streptozotocin-induced type 1 diabetic mice; KK mice (normoglycemic obese mice with impaired glucose tolerance)	BDNF central (ICV) and subcutaneous administration	Antidiabetic effects	[16] Nakagawa, T. et al., 2000
BDNF mutant mice (obese BDNF heterozygous mice)	BDNF central administration (third ventricle)	Transient reversion of eating behaviour and obesity	[17] Kernie, S.G. et al., 2000
Bdnf*^klox/klox^* mice (deficiency in long 3′ UTR Bdnf mRNA/severe obesity development)	Viral expression of long 3′UTR Bdnf mRNA in the hypothalamus (VMH)	Complete rescue of hyperphagic obesity	[32] Liao, G.-Y. et al., 2012
Wild-type mice	Intraperitoneal and ICV administration of glucose after 48h fasting period	Increase in BDNF and TrkB mRNA in VMH	[34] Unger, T.J. et al., 2007
	BDNF central administration (third ventricle)	Neurons activation in hypothalamic appetite-regulating centers	
Bdnf ^2L/2L^ mice	Selectively deletion (viral-mediated) of BDNF alleles in the VMH and DMH	Hyperphagic behavior and obesity	
C57BL/6J mice	ICV leptin administration	Increase in BDNF mRNA in the dorsomedial part of VMH	[35] Komori, T. et al., 2006
Wild-type mice	Injection of a MC4R agonist (MTII) into the dorsal third ventricle after a 44 h fasting period	Increase in BDNF mRNA in the VMH	[36] Xu, B. et al., 2003
Wistar Han rats	Intraparenchymal infusion of BDNF in the DVC	Anorexia and weight loss	[38] Bariohay, B. et al., 2005
	Peripheral leptin injection	Increase in BDNF protein content within the DVC	
Wistar Han rats	BDNF ICV injection into the DVC (4th ventricle)	Reduction in food intake	[39] Bariohay, B. et al., 2009
	ICV delivery of a MC3/4R agonist (MTII) into the DVC (4th ventricle)	Increase in the BDNF protein content in the DVC	
	ICV delivery of a MC3/4R antagonist (SHU9119) into the DVC (4th ventricle)	Decrease in the BDNF protein content in the DVC	
Sprague-Dawley rats	ICV (4th ventricle/hindbrain) BDNF injection	Reduction in cumulative food intake and body weight; increase in core temperature	[40] Spaeth, A.M. et al., 2012
	Intraparenchymal injection of BDNF into the medial nucleus tractus solitarius (mNTS)	Suppression of food intake and body weight	
	Intraparenchymal delivery of BDNF into the mNTS after ANA-12 (specific TrkB receptor antagonist) preadministration	Inhibition of the intake-suppressive effect of BDNF	
	ICV (4th ventricle/hindbrain) leptin injection	Increase in the BDNF protein content within the DVC tissue	
Sprague-Dawley rats	BDNF injection into the PVH	Decrease in feeding and body weight; increase in energy expenditure; UCP1 expression increase in BAT	[41] Wang, C. et al., 2007

**Table 3:** Representative animal studies considering the role of BDNF in thermoregulation and volitional physical activity.

Animal Model	Intervention/Stimuli	Effects	Reference
Bdnf-e1^−/−^ mice	Selective disruption of Bdnf expression from promoter 1	Severe obesity, deficits of BAT-mediated thermogenesis, impairment of body temperature response to cold, down-expression of UCP1 and PCG1α in BAT	[47] You, H. et al., 2020
C57BL/KsJ-db/db mice (obese diabetic mice)	BDNF subcutaneous administration	Increase in body temperature and oxygen consumption	[16] Nakagawa, T. et al., 2000
C57BL/KsJ-db/db mice (obese diabetic mice)	BDNF central (ICV injection) and subcutaneous administration	Increase in UCP1 mRNA and protein in BAT, modulation of energy expenditure	[48] Tsuchida, A. et al., 2001
Diet-induced obese (DIO) C57BL/6 mice	Injection of a rAAV vector expressing the human BDNF gene in the hypothalamus	Reproduction of the effects induced by enriched environment: activation of the brown fat gene program (“browning’’ of WAT) and lean phenotype	[49] Cao, L. et al., 2011
Sprague–Dawley rats	BDNF injection into the VMH	Body weight reduction by decreasing food intake and increasing energy expenditure	[50] Wang, C. et al., 2010
Wistar rats	One week of forced moderate exercise	Improvement in spatial memory, increase in BDNF mRNA expression in the dentate gyrus	[53] Bechara, R.G. et al., 2014
C57BL/6 mice	Long-term physical exercise (4 weeks of treadmill and running wheel exercise)	Hippocampal increase in: (i) BDNF mRNA and protein (ii) synaptic load (iii) TrkB receptor levels in astrocytes	[58] Fahimi, A. et al., 2017
Sprague-Dawley rats	Short exercise period (voluntary exercise paradigm)	Enhancement in spatial learning and memory; increase in the mRNA levels of BDNF, TrkB receptor, synapsin I and CREB	[62] Vaynman, S. et al., 2004
	Injection of a BDNF blocker into the hippocampus before the start of wheel running	Block of exercise benefits (reduction in: mRNA levels of BDNF, TrkB receptor, synapsin I and CREB)	
Muscle-specific BDNF Knockout Mice (BDNF^MKO^)	Muscle injury	Abnormal myogenic differentiation and regeneration	[70] Clow, C.; Jasmin, B.J., 2010
C57BL/KsJ-db/db mice (obese diabetic mice)	Chronic subcutaneous administration of BDNF	Improvement of glucose metabolism: enhancement of glucose utilization in peripheral tissues (skeletal muscle, heart, BAT and liver)	[75] Yamanaka, M. et al., 2007

## Data Availability

Not applicable.
